# Corrigendum

**DOI:** 10.1002/ehf2.13477

**Published:** 2021-06-17

In the paper by Bergh *et al*.,^1^ the 10th author's name has been misspelt.

It should be “Kristjan Karason” as per the corrected author list below:

Niklas Bergh, Einar Gude, Sven‐Erik Bartfay, Arne K Andreassen, Satish Arora, Pia Dahlberg, Göran Dellgren, Lars Gullestad, Finn Gustafsson, Kristjan Karason, Göran Rådegran, Entela Bollano and Bert Andersson

The online version has been modified to reflect the change.
